# The first determination and analysis of the complete mitochondrial genome of *Ancistrus temmincki* (Siluriformes: Loricariidae)

**DOI:** 10.1080/23802359.2020.1866446

**Published:** 2021-05-04

**Authors:** Fang Meng, Xiaolong Yin, Tao Zhang, Chunyan Zhao, Xianglong Xue, Xianglong Xia, Xintao Zhu, Zaixian Duan, Bingjian Liu, Yifan Liu

**Affiliations:** a National Engineering Research Center for Marine Aquaculture, Zhejiang Ocean University, Zhoushan, China; b National Engineering Laboratory of Marine Germplasm Resources Exploration and Utilization, Marine Science and Technology College, Zhejiang Ocean University, Zhoushan, China; c Zhoushan Fisheries Research Institute of Zhejiang Province, Zhoushan, China; d Zhejiang Province Key Lab of Mariculture and Enhancement, Marine Fisheries Research Institute of Zhejiang, Zhoushan, China; e School of Marine Science and Engineering, Qingdao Agricultural University, 266109, Qingdao, China

**Keywords:** *Ancistrus temmincki*, mitochondrial genome, evolutionary relationship

## Abstract

In order to fully comprehend the evolution and kinship of fishes in the family of Loricariidae, the complete mitochondrial genome of the Loricariidae fish *Ancistrus temmincki* was firstly characterized in the present study. The whole mitogenome was 16,657 bp in size and consisted of 13 protein-coding genes, 22 tRNAs, 2 rRNAs genes, a control region and origin of light-strand replication. The proportion of coding sequences with a total length of 11,473 bp was 68.88%, which encoded 3,813 amino acids. The genome composition was highly A + T biased (56.29%), and exhibited AT-skew (0.0661) and a negative GC-skew (–0.2740). All protein-coding genes were started with ATG except for GTG in CO1, while stopped with the standard TAN codons or a single T. The control region (D-loop) ranging from 15,635 bp to 16,657 bp was 1023 bp in size. Until now, there is hardly any studies on the complete mitochondrial sequence in the genus of *Ancistrus*, phylogenetic analysis showed that *A. temmincki* was most closely related to *Ancistrus cryptophthalmus* in the genus of *Ancistrus*. The complete mitochondrial genome sequence has provided a new insight into the taxonomic classification, and a more complex picture of the species diversity within the family of Loricariidae.

*Ancistrus temmincki*, a newly discovered species of fish in the family Loricariidae, now has been initially positioned as *Ancistrus*. It inhabits the turbulent area of the river, adsorbs on the rock, eats the moss on the rock, and has a gentle temperament. Its body is covered with small gray spots and extends to the fins. The head is big and the eyes are behind. There are tentacles on the upper surface of the kiss on the head. The female fish has only one row of bristles between the eyes, while the male fish has two rows of bristles that form a ‘V’ shape (Geerinckx et al. 2007). The body length can reach to 9.8 cm. So far, the reports about this species have limited to the analysis of its appearance (Avise et al. 1987; Xu et al. 2011; Huang et al. 2016). In this study, we firstly determined the complete mitochondrial genome of *A. temmincki*. Additionally, the phylogenetic relationship of Siluriformes (Loricariidae: Ancistrus) was reconstructed based on 13 PCGs for the first time, which involved 15 typical representative species in Perciformes. The present study would supply references to the limited data on molecular level and clarify the classification relationship, even be referenced for systematics.

*Ancistrus temmincki*, a newly discovered species of fish in the family Loricariidae, now has been initially positioned as *Ancistrus*. It inhabits the turbulent area of the river, adsorbs on the rock, eats the moss on the rock, and has a gentle temperament. The fishes collected from the Surin of South America (3°38′38″N, 54°02′06″W). All samples (AT201912) have been deposited in the Zhejiang Engineering Research Center for Mariculture, Zhejiang Ocean University, Zhoushan, China. Tissue samples were reserved in 95% ethanol for molecular analysis and stored at −20 °C. Total genomic DNA was extracted from the muscle using the phenol-chloroform method (Barnett and Larson 2012; Toni et al. 2018). Based on the existing complete mitochondrialgene of *A. cryptophthalmus* (Accession MF804392.1), 12 pairs of primers were designed (Supplementary Table S1) and synthesized by Primer Premier 6.0 (Lalitha 2000). The samples were amplified by Polymerase chain reaction (PCR), PCR was conducted using the following conditions: denaturation at 95 °C for 5 min, 35 amplification cycles (95 °C denaturation for 30 s, 52–55 °C annealing for 30 s, 72 °C extension for 60 s), and a final extension at 72 °C for 10 min. Subsequently, sequences using Sanger sequencing technology and using CodonCode Aligner 5.1.5 (CodonCode Corporation, Dedham, MA) to assemble. The complete mitochondrial genome was annotated using Sequin version 15.10 (http://www.ncbi.nlm.nih.gov/Sequin) and tRNAscan-SE version 2.0 (http://trna.ucsc.edu/tRNAscan-SE/) (Lowe and Eddy 1997). The calculation of base composition and phylogenetic construction was conducted by MEGAX.

Similar to the typical mitogenome of vertebrates, the mitogenome of *A. temmincki* was a closed double-stranded circular molecule of 16,657 bp including 13 protein-coding genes, 2 ribosomal RNA genes, 22 tRNA genes and two main noncoding regions (Boore, 1999; Zhu et al. 2018) deposited in GenBank under accession number MT528234. The proportion of coding sequences with a total length of 11,473 bp was 68.88%, 13 protein-coding genes (PCGs) encode 3813 amino acids in total. The overall contents of A, T, G, and C were 30.01%, 26.28%, 15.87%, and 27.84%, respectively. A-T and G-C contents of mitochondrial genome were 56.29% and 43.71% respectively, thereby with a high AT bias. Both AT-skew and GC-skew of the mitogenome were 0.0661 and −0.2740, respectively. All the protein-coding genes used the initiation codon ATG except for GTG in CO1, which was quite common in vertebrate mtDNA (Miya et al. 2001). Most of them have TAA or TAG as the termination codon, except ND4 used termination condons AGA and CO2 used an incomplete termination codon T. The termination codons observed in the *A. temmincki* genomes were similar to those of most mammals (Kitpipit et al. 2012; Hassanin 2016). Most mitochondrial genes were encoded on the H-strand except for ND6 and eight tRNA genes (Gln, Ala, Asn, Cys, Tyr, Ser, Glu, and Pro), which were encoded on the other complementary strand. The lengths of 12S ribosomal RNA and 16S ribosomal RNA were 951 bp and 1675 bp, which were both located in the typical positions between tRNA-Phe and tRNA-Leu, being separated by tRNA-Val (Petrillo et al. 2006). While the length of control region (D-loop) was 1023 bp, ranging from 15,635 bp to 16,657 bp.

To explore the phylogenetic position of *A. temmincki*, we used a total of 14 species (Supplementary Table S2) of Perciformes mitochondrial genomes, belonging to 8 families. The splicing tandem alignment in the phylogenomic analysis included 13 PCGs after removing the conserved blocks, based on the maximum likelihood (ML) analysis constructed phylogenetic tree. The GenBank accession numbers were listed before the species names. The results of the present study suggested that *A. temmincki* had a closest relationship with *A. cryptophthalmus*, highly supported by a bootstrap value of 100 (Figure 1), which was well consistent with the results based on morphology and other molecular methods. Phylogenetic analysis used to get a clear understanding of classification status, and has well clarified the phylogenetic classification of *A. temmincki* here. Therefore, our findings have provided more details of this species, and would help to the further studies on the family of Loricariidae.

**Figure 1.**
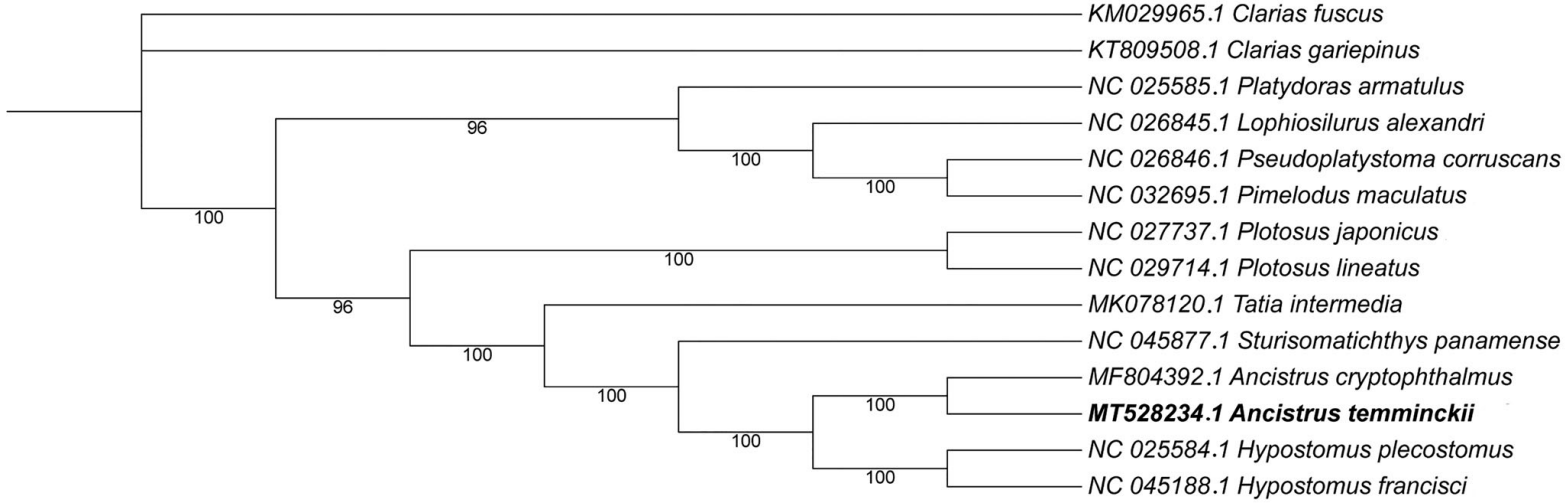
Maximum likelihood (ML) tree of 14 Siluriformes species based on 13 PCGs. The number at each node is the bootstrap probability. The GenBank accession numbers were listed before the species names. The genome sequence in this study has been represented in bold font.

## Supplementary Material

Supplemental Material

## Data Availability

The data that support the findings of this study are openly available in GenBank of NCBI at https://www.ncbi.nlm.nih.gov/, reference number MT528234.

## References

[CIT0001] Avise JC, Arnold J, Ball RM, Bermingham E, Lamb T, Neigel JE, Reeb CA, Saunders NC. 1987. Intraspecific phylogeography: the mitochondrial DNA bridge between population genetics and systematics. Annu Rev Ecol Syst. 18(1):489–522.

[CIT0002] Barnett R, Larson G. 2012. A phenol-chloroform protocol for extracting DNA from ancient samples. Methods Mol Biol. 840:13–19. 22237516 10.1007/978-1-61779-516-9_2

[CIT0003] Boore JL. 1999. Animal mitochondrial genomes. Nucleic Acids Res. 27(8):1767–1780. 10101183 10.1093/nar/27.8.1767 PMC148383

[CIT0004] Geerinckx T, Brunain M, Adriaens D. 2007. Development of the osteocranium in the suckermouth armored catfish *Ancistrus* cf. triradiatus (Loricariidae, siluriformes). J Morphol. 268(3):254–274. 17299777 10.1002/jmor.10515

[CIT0005] Hassanin A. 2016. The complete mitochondrial genome of the African palm civet, Nandinia binotata, the only representative of the family Nandiniidae (Mammalia, Carnivora). Mitochondrial DNA Part A. 27(2):902–904. 10.3109/19401736.2014.926478 24937569

[CIT0006] Huang ZH, Tu FY, Ke DH. 2016. Complete mitochondrial genome of blue-throated bee-eater merops viridis (coraciiformes: meropidae) with its taxonomic consideration. PJZ. 49(1):79–84.

[CIT0007] Kitpipit T, Tobe SS, Linacre A. 2012. The complete mitochondrial genome analysis of the tiger (*Panthera tigris*). Mol Biol Rep. 39(5):5745–5754. 22207170 10.1007/s11033-011-1384-z

[CIT0008] Lalitha S. 2000. Primer Premier 5. Biotech Software Internet Rep. 1(6):270–272.

[CIT0009] Lowe T, Eddy S. 1997. tRNAscan-SE: a program for improved detection of transfer RNA genes in genomic sequence. Nucleic Acids Res. 25(5):955–964. 9023104 10.1093/nar/25.5.955 PMC146525

[CIT0010] Miya M, Kawaguchi A, Nishida M. 2001. Mitogenomic exploration of higher teleostean phylogenies: a case study for moderate-scale evolutionary genomics with 38 newly determined complete mitochondrial DNA sequences. Mol Biol Evol. 18(11):1993–2009. 11606696 10.1093/oxfordjournals.molbev.a003741

[CIT0011] Petrillo M, Silvestro G, Di Nocera PP, Boccia A, Paolella G. 2006. Stem-loop structures in prokaryotic genomes. BMC Genom. 7:170. 10.1186/1471-2164-7-170 PMC1590033 16820051

[CIT0012] Toni LS, Garcia AM, Jeffrey DA, Jiang X, Stauffer BL, Miyamoto SD, Sucharov CC. 2018. Optimization of phenol-chloroform RNA extraction. MethodsX. 5:599–608. 29984193 10.1016/j.mex.2018.05.011 PMC6031757

[CIT0013] Xu K, Kanno M, Yu H, Li Q, Kijima A. 2011. Complete mitochondrial DNA sequence and phylogenetic analysis of Zhikong scallop *Chlamys farreri* (Bivalvia: Pectinidae). Mol Biol Rep. 38(5):3067–3074. 20131010 10.1007/s11033-010-9974-8

[CIT0014] Zhu K, Gong L, Jiang L, Liu L, Lü Z, Liu B-j. 2018. Phylogenetic analysis of the complete mitochondrial genome of *Anguilla japonica* (Anguilliformes, Anguillidae). Mitochondrial DNA Part B. 3(2):536–537. 33474232 10.1080/23802359.2018.1467225 PMC7799773

