# Supplementary material for: The first determination and analysis of the complete mitochondrial genome of Ancistrus temmincki (Siluriformes: Loricariidae)
Source: Mitochondrial DNA B Resour. 2021 May 4;6(5):1583–5. doi: 10.1080/23802359.2020.1866446 (PMC8110184; doi:10.1080/23802359.2020.1866446)
Supplement: Supplemental Material [file TMDN_A_1866446_SM8109.pdf]

Supplementary Table S1. Sequences of the primers used in this study

Primer name	Primer sequence (5′−3′)
F70	CACAAAGGCTTGGTCCTGACT
R1270	CAGCGTTCCCTTGCGGTACTT
F1033	ACATGGTAAGTGTACCGGAAGG
R2715	CTGGATTACTCCGGTCTGA
F2319	AAGACGAGAAGACCCTATGGAG
R3668	GAADGGBCCYCCNGCRTAYTCTAC
F3410	GCCTGACCACTAGCTGCAAT
R5295	CTGGCTTGGGCGCTTAGCTGT
F5118	AGACCRAGGGCCTTCAAAG
R7684	TARCTTCARTATCATTGRTGKCC
F6763	GTAGTAGCMCACTTYCACTAYGT
R8952	TGTGCTTGGTGGGCCATT
F8473	AACCHRGGVGGNCAYAARTGAGC
R9751	GATCCTCATCARTAGATDGAGAC
F9685	TTYGAAGCNGCYGCCTGATACTGACAYTT
R11430	GGTTCGGCTGTGGGTTCGTTC
F10531	CTACAATGCTAAAARTYCTHATCCC
R12110	TAGCCYHTGCTTGGATTTGCACCAAGAGT
F12085	GAAGGATAAYAGTCATCCGYTGGTCTTAGG
R14480	CTACTGCGTCGTTGGCGATTTT
F13400	ATCAARCGACTTGCCTGAGGAAG
R15850	ATAGTTTAANTTBAGAATNCTAGCTTTGGG
F15673	CTARCTCCCAAAGCTAGNATTCT
R201	CACTGCTGAATTCCCTTGGGGGTGTG

Supplementary Table S2. List of organisms used in the phylogenetic analyses.

**Species**	**Family**	**Size (bp)**	**GeneBank No**
Tatia intermedia	Auchenipteridae	16582	MK078120.1
Clarias fuscus	Clariidae	16525	KM029965.1
Clarias gariepinus	Clariidae	16505	KT809508.1
Lophiosilurus alexandri	Pseudopimelodidae	16445	NC_026845.1
Pseudoplatystoma corruscans	Pimelodidae	16123	NC_026846.1
Pimelodus maculatus	Pimelodidae	16561	NC_032695.1
Hypostomus plecostomus	Loricariidae	16523	NC_025584.1
Plotosus japonicus	Plotosidae	16559	NC_027737.1
Plotosus lineatus	Plotosidae	16480	NC_029714.1
Platydoras armatulus	Doradidae	16470	NC_025585.1
Ancistrus cryptophthalmus	Loricariidae	16422	MF804392.1
Ancistrus temmincki	Loricariidae	16657	MT528234.1

For Peer Review Only
